# Dopamine receptor D_2_ activation suppresses the radiosensitizing effect of aripiprazole via activation of AMPK

**DOI:** 10.1002/2211-5463.12699

**Published:** 2019-07-23

**Authors:** Hyounji Lee, Seongman Kang, Jong Kyung Sonn, Young‐Bin Lim

**Affiliations:** ^1^ Division of Radiation Biomedical Research Korea Institute of Radiological and Medical Sciences Seoul Korea; ^2^ Division of Life Science, College of Life Sciences and Biotechnology Korea University Seoul Korea; ^3^ Department of Biology, College of Natural Sciences Kyungpook National University Daegu Korea

**Keywords:** AMPK, breast cancer, dopamine receptor, drug repositioning, radiotherapy

## Abstract

Drug repositioning has garnered attention as an alternative strategy to the discovery and development of novel anticancer drug candidates. In this study, we screened 321 FDA‐approved drugs against nonirradiated and irradiated MCF‐7 cells, revealing that aripiprazole, a dopamine receptor D2 (D2R) partial agonist, enhances the radiosensitivity of MCF‐7 cells. Unexpectedly, D2R‐selective antagonist treatment significantly enhanced the radiosensitizing effects of aripiprazole and prevented aripiprazole‐induced 5' adenosine monophosphate‐activated protein kinase (AMPK) phosphorylation. Direct AMPK activation with A769662 treatment blunted the radiosensitizing effects of aripiprazole. These results indicate that aripiprazole has potential as a radiosensitizing drug. Furthermore, prevention of D2R/AMPK activation might enhance these anticancer effects of aripiprazole in breast cancer cells.

**Abbreviations**
ACC: acetyl‐coenzyme A carboxylase
AMPK: 5' adenosine monophosphate‐activated protein kinase
D2R: dopamine receptor D_2_
PARP: poly (ADP‐ribose) polymerase 1
siRNA: small interfering RNA

Several success stories of drug repositioning have received global attention in recent years. Drug repositioning refers to the identification of new indications for existing FDA‐approved and abandoned drugs, due to issues other than drug safety for treating diseases other than the disease for which the drug was intended 1. The traditional approach to drug discovery, development, and registration is a time‐consuming and costly process, and 70–90% of drugs fail clinical trials. This expensive and time‐consuming process of discovering and developing new drugs calls for alternative approaches such as drug repositioning 1, 2. Since drug repositioning involves an evaluation of potential new uses for existing drugs that have already been used in humans, it affords a reduced length and cost of the research and trial phase by utilizing existing data, such as the drug's dose regimen with favorable pharmacokinetic and pharmacodynamic properties as well as tolerable side effects. One of the well‐known examples of drug repositioning is the use of sildenafil (Viagra) for erectile dysfunction. Sildenafil was originally developed for the treatment of coronary artery disease by Pfizer in the 1980s 3. Drug repositioning also has attracted attention as a powerful alternative strategy to the discovery and development of novel anticancer drug candidates with the successful clinical introduction of a number of noncancer drugs for cancer treatment 4.

Breast cancer is one of the leading causes of cancer‐related death among women worldwide 5. Radiotherapy is one of the mainstay treatments of local control of breast cancer and is utilized in the majority of patients 6. Although randomized trials have demonstrated the efficacy of radiation therapy in the treatment of breast cancer, the rates of recurrence and metastasis in breast cancer are substantial 7, 8, 9, 10. The recent wealth of research has suggested that biological factors play a role in determining the efficacy of radiation therapy. The biological factors of tumors that affect outcome after radiotherapy include the intrinsic radioresistance of tumor cells 11, 12. The PI3K‐AKT, nuclear factor‐κB, and mitogen‐activated protein kinase pathways can mediate the intrinsic radioresistance of tumor cells and are often aberrantly activated in tumors. Multimodal treatment strategies combining pharmacological interventions targeting these pathways with ionizing radiation (IR) are therefore promising for increasing the radiosensitivity of cancer cells, which might lead to the improvements in local cancer control and patient survival. Therefore, in this study, we screened a library of FDA‐approved drugs to identify compounds that radiosensitize breast cancer cells. Of the 321 compounds in the library, aripiprazole was found to show a selective cytotoxic effect on irradiated breast cancer cells. Aripiprazole was subjected to further analysis to evaluate the mechanism underlying its radiosensitizing effects.

## Materials and methods

### Cell culture

The human breast cancer cell lines MCF‐7 and BT‐474 were cultured in Dulbecco's modified Eagle's medium and RPMI 1640 supplemented with 10% heat‐inactivated FBS (Lonza, Rockland, ME, USA), respectively. Both cell lines were grown at 37 °C in a humidified atmosphere with 5% CO_2_ and were in the logarithmic growth phase at the initiation of experiments.

### Materials and irradiation

Aripiprazole, quinpirole, and haloperidol were purchased from Tocris (Ellisville, MO, USA). A769662 was obtained from LC Laboratories (Woburn, MA, USA). Thioridazine was obtained from Cayman Chemical (Ann Arbor, MI, USA). Irradiation was performed at room temperature by using a ^137^Cs gamma‐ray source Gammacell 3000 manufactured by Nordion (Ottawa, ON, Canada).

### Compound library

A library of FDA‐approved compounds (catalog number BML‐2843) was purchased from Enzo Life Sciences (Plymouth Meeting, PA, USA) to screen compounds that increase the radiosensitivity of MCF‐7 breast cancer cells.

### Screening with nonirradiated and irradiated MCF‐7 cells

MCF‐7 cells were seeded in 96‐well plates at a density of 1 × 10^3^ cells per well. Twenty‐four hours later, the cells were pretreated with compounds at a concentration of 5 µm for 1 h and subsequently treated with or without 5 Gy of IR. Ninety‐six hours later, cell viability was determined using an EZ‐Cytox Cell Viability Assay Kit (water‐soluble tetrazolium salt method), according to the manufacturer’s protocol (DOGEN, Seoul, Korea). Percent viability was calculated relative to that of the cells in DMSO‐treated control wells.

### Western blotting

Total cell lysates were prepared using RIPA buffer, and western blot analyses were performed as previously described 13. Anti‐poly (ADP‐ribose) polymerase 1 (PARP) (catalog number 9542), phospho‐5' adenosine monophosphate‐activated protein kinase (AMPK)α (catalog number 2535), AMPK (catalog number 2532), phospho‐acetyl‐coenzyme A carboxylase (ACC) (catalog number 3661), and ACC (catalog number 3676) antibodies were purchased from Cell Signaling Technology (Danvers, MA, USA).

### Small interfering RNA and cell transfection

Small interfering RNA (siRNA) oligonucleotides were designed using the siRNA design tool provided by Dharmacon Research (Lafayette, CO, USA). The oligonucleotide sequences were as follows: 5′‐CAACUAUGCUGCACCAGAAGTAA‐dTdT‐3′ (*PRKAA1* siRNA) and 5′‐AAAUGAACGUGAAUUGCUCAA‐dTdT‐3′ (luciferase siRNA). Transfection was performed using the RNAiMAX protocol provided by Invitrogen (Carlsbad, CA, USA). After 24 h, the transfected cells were used for experiments.

### DNA fragmentation assay

Internucleosomal DNA fragmentation was quantitatively determined by using the Cell Death Detection ELISA^PLUS^ Kit (Roche, Mannheim, Germany), according to the manufacturer’s instructions, as previously described 14. Briefly, cells were seeded in 96‐well plates at a density of 5 × 10^3^ cells per well and then treated with IR. Forty hours after IR treatment, the cells were resuspended in 200 µL of the lysis buffer that was supplied by the manufacturer. After incubating for 30 min at room temperature, 20 µL of the extract was used in the enzyme‐linked immunosorbent assay, according to the manufacturer’s protocol. Finally, upon incubating with a peroxidase substrate for 15 min, the absorbance at 405 nm with a reference wavelength of 492 nm was determined with a microplate reader (Bio‐Tec Instruments, Winooski, VT, USA).

### Statistical analysis

The results were expressed as the mean ± standard deviation (SD) values of three to four experiments that were performed independently. The normality distribution of the variables was assessed using the Kolmogorov–Smirnov test. The paired Student’s *t*‐test was performed where indicated, and *P*‐values of less than 0.05 were considered statistically significant.

## Results

### Identification of FDA‐approved drugs that regulated the radiosensitivity of breast cancer cells

**Figure 1:**
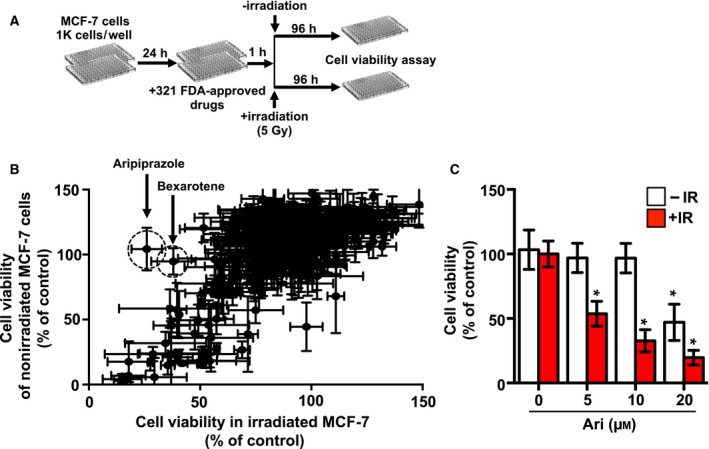
Screening for FDA‐approved drugs that radiosensitize breast cancer cells identifies aripiprazole. (A) Schematic representation of differential screening with nonirradiated and irradiated MCF‐7 cells. (B) XY scatter plot of percent viability of the cells treated with the 321 compounds selected for screening. Regions circled with dash lines indicate compounds that reduced cell viability to less than 50%. (C) MCF‐7 cells were pretreated with indicated concentrations of aripiprazole and subsequently treated with or without 5 Gy of IR. Ninety‐six hours later, cell viability was determined using an EZ‐Cytox Cell Viability Assay Kit. Data represent means ± SD of three independent experiments (Student’s *t*‐test; *P < 0.05).

To identify compounds that radiosensitize breast cancer cells, we screened 321 FDA‐approved drugs against nonirradiated and irradiated MCF‐7 cells at a concentration of 10 µm (Fig. 1A). We defined the primary hits as drugs that reduce cell viability to less than 50% of the control cells. We selected irradiated cell‐selective cytotoxic drugs as radiosensitizing drugs, and nonselective cytotoxic drugs that equally targeted nonirradiated cells were excluded from further evaluation, although these drugs were known or potential anticancer candidates (Fig. 1B). Of the 321 compounds screened, two drugs were found to show a selective effect on irradiated MCF‐7 cells compared to that on nonirradiated MCF‐7 cells. These two irradiated MCF‐7 cell‐selective drugs were aripiprazole and bexarotene. Bexarotene is already known to radiosensitize cells and was excluded from further evaluation 15, 16. To validate the radiosensitizing effects of aripiprazole, we retested its cytotoxicity toward nonirradiated and irradiated MCF‐7 cells in a dose–response series. As shown in Fig. 1C, aripiprazole enhanced the radiosensitivity of MCF‐7 cells in a dose‐dependent manner. Interestingly, 20 µm aripiprazole treatment reduced the viability of MCF‐7 cells, whereas 5 or 10 µm aripiprazole caused no alterations in cell viability.

### Activation of dopamine receptor D_2_ was not necessary for aripiprazole‐induced apoptosis in breast cancer cells

**Figure 2:**
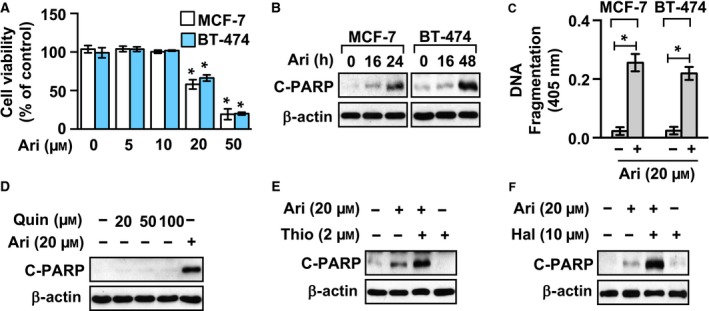
Treatment with a D2R‐selective antagonist augments aripiprazole‐induced apoptosis in human breast cancer cells. (A) Cells were treated with indicated concentrations of aripiprazole for 48 h. Cell viability was then determined using an EZ‐Cytox Cell Viability Assay Kit. Data represent means ± SD of three independent experiments (Student’s *t*‐test; *P < 0.05). (B) Cleavage of PARP was examined by western blotting at the indicated time points after treatment with 20 µm aripiprazole. (C) Cells were treated with 20 µm aripiprazole for 40 h. Internucleosomal DNA fragmentation was then measured using the Cell Death Detection ELISA^PLUS^ Kit (Roche). Data represent means ± SD of three independent experiments (Student’s *t*‐test; *P < 0.05). (D) MCF‐7 cells were treated with 20 µm aripiprazole or indicated concentrations of quinpirole for 24 h. Cleavage of PARP was then examined by western blotting. (E) MCF‐7 cells were pretreated for 1 h with or without 2 µm thioridazine and subsequently treated with or without 20 µm aripiprazole for 16 h. Cleavage of PARP was then determined by western blotting. (F) MCF‐7 cells were pretreated with or without 10 µm haloperidol for 1 h and subsequently treated with or without 20 µm aripiprazole. Cleavage of PARP was then determined by western blotting.

The anticancer activity of aripiprazole in MCF‐7 cells was also observed in BT‐474 cells, which express exon 8‐mutant p53, indicating the anticancer activity of aripiprazole is not specific to MCF‐7 cells (Fig. 2A). The breast cancer cells became round and detached upon treatment with aripiprazole (data not shown). Cancer cells undergoing apoptosis usually become round; therefore, we measured poly (ADP‐ribose) polymerase (PARP, substrate of caspase‐3 and caspase‐7) cleavage to determine whether aripiprazole induced apoptosis in the cells. As shown in Fig. 2B, 20 µm aripiprazole treatment induced PARP cleavage in breast cancer cells. In addition, we observed apoptotic DNA fragmentation in the cells treated with aripiprazole (Fig. 2C). We then compared the responses to treatment with quinpirole, a dopamine receptor D_2_ (D2R)‐specific agonist, with those to treatment with aripiprazole in MCF‐7 cells to determine whether the underlying mechanism of aripiprazole‐induced apoptosis in breast cancer cells involved D2R signaling. As shown in Fig. 2D, 20 µm aripiprazole treatment induced significant PARP cleavage. On the other hand, quinpirole treatment up to 100 µm showed no detectable expression of cleaved PARP, suggesting that the aripiprazole‐induced apoptosis in the breast cancer cells was unlikely to be mediated via activation of D2R signaling. Surprisingly, pretreatment with thioridazine, a representative D2R‐selective antagonist, significantly enhanced aripiprazole‐induced PARP cleavage (Fig. 2E). Similar to thioridazine, haloperidol, another D2R‐selective antagonist, also resulted in enhancement of aripiprazole‐induced PARP cleavage (Fig. 2F), suggesting that D2R activation did not contribute to aripiprazole‐induced apoptosis in human breast cancer cells. However, aripiprazole‐induced D2R signaling nullified the aripiprazole‐induced apoptosis.

### D2R‐mediated AMPK activation suppressed aripiprazole‐induced apoptosis

**Figure 3:**
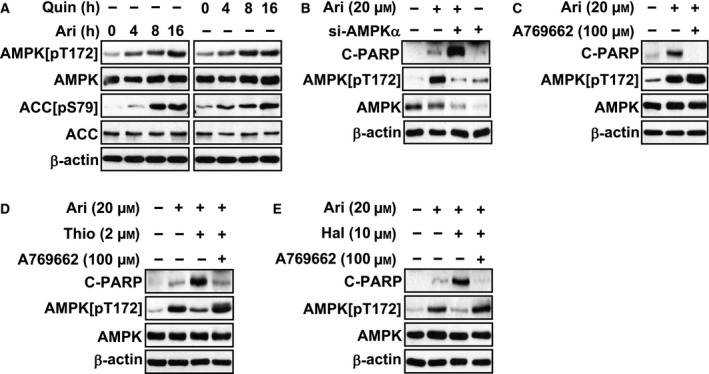
Treatment with A769662, a direct AMPK activator, blunts the enhancing effects of D2R‐selective antagonists on aripiprazole‐induced apoptosis. (A) Phosphorylation of AMPK and ACC was examined by western blotting at the indicated time points after treatment with 20 µm aripiprazole or 20 µm quinpirole in MCF‐7 cells. (B) Twenty‐four hours after transfection with luciferase siRNA (control) or AMPKα siRNA (siAMPK), MCF‐7 cells were treated with or without 20 µm aripiprazole for 16 h. Cleavage of PARP and phosphorylation of AMPK were then determined by western blotting. (C) MCF‐7 cells were pretreated for 1 h with or without 100 µm A769662 and subsequently treated with 20 µm aripiprazole for 16 h. Cleavage of PARP and phosphorylation of AMPK were then determined by western blotting. (D) MCF‐7 cells were pretreated for 1 h with or without 100 µm A769662 and subsequently treated with or without 2 µm thioridazine for another 1 h. Cleavage of PARP and phosphorylation of AMPK were then determined by western blotting at 16 h after 20 µm aripiprazole treatment in the MCF‐7 cells. (E) MCF‐7 cells were pretreated for 1 h with or without 100 µm A769662 and subsequently treated with or without 10 µm haloperidol for another 1 h. Cleavage of PARP and phosphorylation of AMPK were then determined by western blotting at 16 h after 20 µm aripiprazole treatment in the MCF‐7 cells.

Given that no significant changes were observed in aripiprazole‐induced apoptosis in MCF‐7 cells pretreated with dibutyryl‐cAMP, an analog of cAMP that stimulates protein kinase A (data not shown), we next examined whether this antiapoptotic function of D2R activation during aripiprazole‐induced apoptosis involved the AMPK pathway in breast cancer cells. As shown in Fig. 3A, aripiprazole treatment activated AMPK pathway, as indicated by increased AMPK and ACC phosphorylation in response to aripiprazole treatment. Quinpirole also induced phosphorylation of AMPK and ACC, suggesting that the ability of aripiprazole to activate D2R contributed to the activation of the AMPK pathway in response to aripiprazole treatment in MCF‐7 cells. We next silenced AMPK with siRNA to examine the possible involvement of the AMPK pathway in aripiprazole‐induced apoptosis in MCF‐7 cells. AMPK knockdown in MCF‐7 cells led to increased PARP cleavage compared to that in MCF‐7 cells pretreated with control siRNA (Fig. 3B). On the contrary, treatment with A769662, a direct AMPK activator, completely prevented the aripiprazole‐induced apoptosis (Fig. 3C). The increased apoptotic resistance mediated by D2R‐mediated AMPK activation during the aripiprazole‐induced apoptosis suggests that prevention of D2R/AMPK activation in response to aripiprazole treatment might be a strategy to enhance the anticancer effects of aripiprazole in breast cancer cells. To confirm this hypothesis, we used thioridazine. Thioridazine treatment prevented aripiprazole‐induced AMPK phosphorylation and enhanced aripiprazole‐induced PARP cleavage (Fig. 3D). Haloperidol also had the same effects (Fig. 3E). Direct AMPK activation with A769662 treatment blunted the enhancing effects of D2R antagonists on aripiprazole‐induced apoptosis (Fig. 3D,E). Overall, these findings suggest a nullifying contribution of D2R‐mediated AMPK activation on the anticancer effects of aripiprazole.

### D2R‐mediated AMPK activation suppressed the radiosensitizing effects of aripiprazole in human breast cancer cells

**Figure 4:**
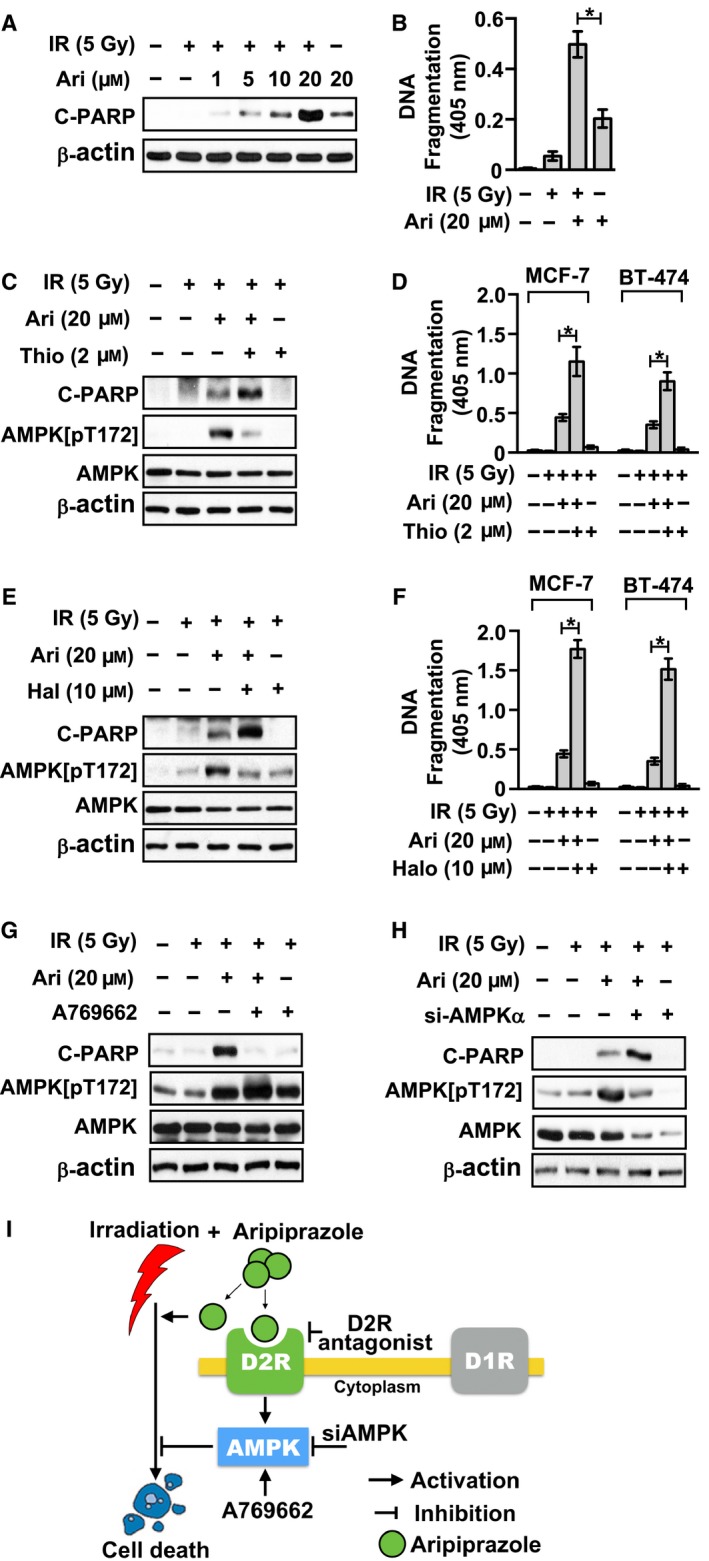
D2R‐selective antagonists augment the radiosensitizing effects of aripiprazole in human breast cancer cells. (A) MCF‐7 cells were pretreated for 1 h with or without the indicated concentration of aripiprazole. Cleavage of PARP was then determined by western blotting at 24 h after treatment of the MCF‐7 cells with 5 Gy of IR. (B) MCF‐7 cells were pretreated for 1 h with or without 20 µm aripiprazole. Internucleosomal DNA fragmentation was then measured using the Cell Death Detection ELISA^PLUS^ Kit (Roche) at 40 h after treatment of the MCF‐7 cells with 5 Gy of IR. Data represent means ± SD of three independent experiments (Student’s *t*‐test; *P < 0.05). (C) MCF‐7 cells were pretreated for 1 h with or without 2 µm thioridazine and subsequently treated with or without 20 µm aripiprazole for another 1 h. Cleavage of PARP and phosphorylation of AMPK were then determined by western blotting at 16 h after treatment of the MCF‐7 cells with 5 Gy of IR. (D) Cells were pretreated for 1 h with or without 2 µm thioridazine and subsequently treated with or without 20 µm aripiprazole for another 1 h. Internucleosomal DNA fragmentation was then measured by using Cell Death Detection ELISA^PLUS^ Kit (Roche) at 40 h after treatment of the cells with 5 Gy of IR. Data represent means ± SD of three independent experiments (Student’s *t*‐test; *P < 0.05). (E) MCF‐7 cells were pretreated for 1 h with or without 10 µm haloperidol and subsequently treated with or without 20 µm aripiprazole for another 1 h. Cleavage of PARP and phosphorylation of AMPK were then determined by western blotting at 16 h after treatment of the MCF‐7 cells with 5 Gy of IR. (F) Cells were pretreated for 1 h with or without 10 µm haloperidol and subsequently treated with or without 20 µm aripiprazole for another 1 h. Internucleosomal DNA fragmentation was then measured using Cell Death Detection ELISA^PLUS^ Kit (Roche) at 40 h after treatment of the MCF‐7 cells with 5 Gy of IR. Data represent means ± SD of three independent experiments (Student’s *t*‐test; *P < 0.05). (G) MCF‐7 cells were pretreated for 1 h with or without 100 µm A769662 and subsequently treated with 20 µm aripiprazole for another 1 h. Cleavage of PARP and phosphorylation of AMPK were then determined by western blotting at 16 h after treatment of the MCF‐7 cells with 5 Gy of IR. (H) Twenty‐four hours after transfection with luciferase siRNA (control) or AMPKα siRNA (siAMPK), MCF‐7 cells were pretreated for 1 h with or without 20 µm aripiprazole. Cleavage of PARP and phosphorylation of AMPK were then determined by western blotting at 16 h after treatment of the MCF‐7 cells with 5 Gy of IR. (I) Schema showing the nullifying contribution of D2R/AMPK pathway to the radiosensitizing effects of aripiprazole in breast cancer cells.

We sought to determine whether aripiprazole could also regulate apoptosis in IR‐exposed breast cancer cells. As shown in Fig. 4A, aripiprazole treatment led to increased PARP cleavage in IR‐exposed MCF‐7 cells compared to that in the mock‐irradiated MCF‐7 cells. In addition, DNA fragmentation markedly increased in irradiated cells in response to aripiprazole treatment compared to that in the mock‐irradiated cells (Fig. 4B). Interestingly, thioridazine treatment significantly enhanced the radiosensitizing effects of aripiprazole in MCF‐7 cells, as indicated by increased PARP cleavage and the significant difference in the level of DNA fragmentation between breast cancer cells with and without thioridazine treatment in response to aripiprazole treatment and following irradiation (Fig. 4C,D). As shown in Fig. 4E,F, the radiosensitizing effects of thioridazine toward the breast cancer cells treated with aripiprazole were replicated by another D2R antagonist haloperidol, which clearly indicated that the D2R pathway did not contribute to the radiosensitizing effects of aripiprazole against breast cancer cells, but rather suppressed them.

As shown in Fig. 4C,E, the prevention of aripiprazole‐induced AMPK activation observed in the cells treated with D2R antagonists raised the possibility that the D2R/AMPK pathway antagonizes the radiosensitizing effects of aripiprazole against breast cancer cells. To evaluate this possibility, we examined whether direct AMPK activation with A769662 could ameliorate aripiprazole‐induced radiosensitization in MCF‐7 cells. As shown in Fig. 4G, A769662 treatment led to an increase in aripiprazole‐induced AMPK phosphorylation, which was accompanied by the suppression of aripiprazole‐induced PARP cleavage in IR‐exposed MCF‐7 cells. Furthermore, AMPK knockdown in MCF‐7 cells resulted in significantly increased PARP cleavage in IR‐exposed MCF‐7 cells in response to aripiprazole treatment (Fig. 4H). Collectively, these results suggest that the D2R/AMPK pathway nullified the radiosensitizing effects of aripiprazole toward breast cancer cells (Fig. 4I).

## Discussion

Dopamine as a neurotransmitter regulates a wide variety of physiological functions in the central nervous system. Apart from its action as a neurotransmitter, dopamine also exerts a variety of cardiovascular, renal, and immunological effects 17. Dopamine exerts its action by binding to specific membrane receptors that belong to the G protein‐coupled receptor superfamily. Dopamine receptors can be divided as either D1‐like receptors (D1 and D5) or D2‐like receptors (D2–D4) based on their effects on the accumulation of cyclic AMP (cAMP). D1‐like receptors generally induce an increase in intracellular cAMP. In contrast, D2‐like receptors inhibit intracellular cAMP accumulation on stimulation 18. Recently, the expression of dopamine receptors in different human cancer cell lines and the possible role of dopamine receptor signaling in the control of growth and differentiation of cancer cells have been described. For example, SKF‐38393 (an agonist of D1R) and thioridazine (an antagonist of D2R) were reported to inhibit the proliferation of acute myeloid leukemia stem cells 19. In fact, thioridazine also shows potent anticancer effects against a wide spectrum of cancer cells including glioblastoma, renal, cervical, and endometrial cancer cell lines 19, 20, 21, 22, suggesting that D2R signaling contributes to the proliferation and maintenance of some cancer cells.

Aripiprazole, developed as a D2R partial agonist, is a widely used antipsychotic drug with fewer side effects, including extrapyramidal symptoms and metabolic disorder, than those associated with other antipsychotic drugs 23, 24. Recently, anticancer activity of aripiprazole has been reported in a series of human cancer cells. At concentrations nontoxic to normal cells, aripiprazole treatment exerted cytotoxic activity in cancer cells by suppressing Src activation, and treatment of cancer cells with a combination of aripiprazole and chemotherapeutic agents, such as fluorouracil (5‐FU), gemcitabine, and cisplatin, caused enhanced cell death compared to that associated with either treatment alone 25, 26. Concerning the prosurvival contributions of D2R signaling in cancer cells 19, 20, 21, 22, the anticancer effects of aripiprazole were totally unexpected.

In this study, screening for FDA‐approved drugs by using breast cancer cells led to the identification of aripiprazole as a new radiosensitizing drug, and induction of apoptosis was observed in response to aripiprazole treatment in breast cancer cells. The apoptosis‐inducing effect of aripiprazole was not replicated by another D2R agonist, quinpirole, suggesting that aripiprazole might not require D2R activation‐dependent molecular mechanisms to exert its unique anticancer activities. Indeed, pretreatment with D2R‐specific antagonists did not prevent the apoptosis‐inducing effect of aripiprazole but rather strengthened it, clearly demonstrating that D2R signaling is not responsible for the aripiprazole‐induced apoptosis in breast cancer cells. It has previously been reported that treating mice with haloperidol reduces tau phosphorylation via D2 blockade‐mediated inactivation of AMPK 27. In this study, breast cancer cells activate the AMPK pathway in response to aripiprazole treatment. Furthermore, quinpirole treatment also activated the AMPK pathway in breast cancer cells, suggesting that AMPK activation in response to aripiprazole treatment in breast cancer is the consequence of D2R occupancy by aripiprazole. In addition, treatment with AMPK‐targeting siRNA significantly increases aripiprazole‐induced PARP cleavage, whereas direct AMPK activation with A769662 completely prevents aripiprazole‐induced PARP cleavage. These data support the nullifying contribution of the D2R/AMPK pathway to the anticancer effects of aripiprazole. The nullifying contribution of D2R/AMPK pathway is also involved in the mechanism underlying aripiprazole‐induced radiosensitization in breast cancer cells. Thus, our findings warrant further experimental and clinical approval for aripiprazole alone or in combination with D2R antagonists as a radiosensitizer for breast cancer treatment.

## Conflict of interest

The authors declare no conflict of interest.

## Author contributions

HL SK, JS, and YL conceived the study; HL performed experiments; HL, SK, JS, and YL wrote the manuscript.

## References

[1] Chong CR and Sullivan DJ (2007) New uses for old drugs. Nature 448, 645–646. 17687303 10.1038/448645a

[2] Weir SJ , DeGennaro LJ and Austin CP (2012) Repurposing approved and abandoned drugs for the treatment and prevention of cancer through public‐private partnership. Cancer Res 72, 1055–1058. 22246671 10.1158/0008-5472.CAN-11-3439 PMC3341848

[3] Boolell M , Allen MJ , Ballard SA , Gepi‐Attee S , Muirhead GJ , Naylor AM , Osterloh IH and Gingell C (1996) Sildenafil: an orally active type 5 cyclic GMP‐specific phosphodiesterase inhibitor for the treatment of penile erectile dysfunction. Int J Impot Res 8, 47–52. 8858389

[4] Pessetto ZY , Weir SJ , Sethi G , Broward MA and Godwin AK (2013) Drug repurposing for gastrointestinal stromal tumor. Mol Cancer Ther 12, 1299–1309. 23657945 10.1158/1535-7163.MCT-12-0968 PMC3707936

[5] Mendes D , Alves C , Afonso N , Cardoso F , Passos‐Coelho JL , Costa L , Andrade S and Batel‐Marques F (2015) The benefit of HER2‐targeted therapies on overall survival of patients with metastatic HER2‐positive breast cancer–a systematic review. Breast Cancer Res 17, 140. 26578067 10.1186/s13058-015-0648-2 PMC4650834

[6] Delaney G , Barton M and Jacob S (2003) Estimation of an optimal radiotherapy utilization rate for breast carcinoma. Cancer 98, 1977–1986. 14584082 10.1002/cncr.11740

[7] Frassica DA and Zellars R (2002) Radiation oncology: the year in review. Curr Opin Oncol 14, 594–599. 12409648 10.1097/00001622-200211000-00002

[8] Zellars R and Frassica D (2001) Radiation therapy in the management of breast cancer: an annual review of selected publications. Curr Opin Oncol 13, 431–435. 11673682 10.1097/00001622-200111000-00004

[9] Asrari F and Gage I (1999) Radiation therapy in management of breast cancer. Curr Opin Oncol 11, 463–467. 10550009 10.1097/00001622-199911000-00006

[10] Gage I and Harris JR (1998) Radiation therapy and breast cancer. Curr Opin Oncol 10, 513–516. 9818229 10.1097/00001622-199811000-00006

[11] Chakravarthy A , Nicholson B , Kelley M , Beauchamp D , Johnson D , Frexes‐Steed M , Simpson J , Shyr Y and Pietenpol J (2000) A pilot study of neoadjuvant paclitaxel and radiation with correlative molecular studies in stage II/III breast cancer. Clin Breast Cancer 1, 68–71. 11899393 10.3816/CBC.2000.n.007

[12] Skinner KA , Silberman H , Florentine B , Lomis TJ , Corso F , Spicer D and Formenti SC (2000) Preoperative paclitaxel and radiotherapy for locally advanced breast cancer: surgical aspects. Ann Surg Oncol 7, 145–149. 10761794 10.1007/s10434-000-0145-3

[13] Lim YB , Kang SS , Park TK , Lee YS , Chun JS and Sonn JK (2000) Disruption of actin cytoskeleton induces chondrogenesis of mesenchymal cells by activating protein kinase C‐alpha signaling. Biochem Biophys Res Commun 273, 609–613. 10873653 10.1006/bbrc.2000.2987

[14] Lee ES , Lee H‐J , Lee Y‐J , Jeong J‐H , Kang S and Lim Y‐B (2014) Chemical chaperones reduce ionizing radiation‐induced endoplasmic reticulum stress and cell death in IEC‐6 cells. Biochem Biophys Res Commun 450, 1005–1009. 24973711 10.1016/j.bbrc.2014.06.091

[15] Apisarnthanarax N , Ha CS and Duvic M (2003) Mycosis fungoides with follicular mucinosis displaying aggressive tumor‐stage transformation : successful treatment using radiation therapy plus oral bexarotene combination therapy. Am J Clin Dermatol 4, 429–433. 12762834 10.2165/00128071-200304060-00006

[16] Yen W‐C and Lamph WW (2005) The selective retinoid X receptor agonist bexarotene (LGD1069, Targretin) prevents and overcomes multidrug resistance in advanced breast carcinoma. Mol Cancer Ther 4, 824–834. 15897247 10.1158/1535-7163.MCT-05-0018

[17] Cherubini E , Di Napoli A , Noto A , Osman GA , Esposito MC , Mariotta S , Sellitri R , Ruco L , Cardillo G , Ciliberto G *et al* (2016) Genetic and functional analysis of polymorphisms in the human dopamine receptor and transporter genes in small cell lung cancer. J Cell Physiol 231, 345–356. 26081799 10.1002/jcp.25079

[18] Beaulieu JM and Gainetdinov RR (2011) The physiology, signaling, and pharmacology of dopamine receptors. Pharmacol Rev 63, 182–217. 21303898 10.1124/pr.110.002642

[19] Sachlos E , Risueño RM , Laronde S , Shapovalova Z , Lee JH , Russell J , Malig M , McNicol JD , Fiebig‐ Comyn A , Graham M *et al* (2012) Identification of drugs including a dopamine receptor antagonist that selectively target cancer stem cells. Cell 149, 1284–1297. 22632761 10.1016/j.cell.2012.03.049

[20] Kang S , Dong SM , Kim BR , Park MS , Trink B , Byun HJ and Rho SB (2012) Thioridazine induces apoptosis by targeting the PI3K/Akt/mTOR pathway in cervical and endometrial cancer cells. Apoptosis 17, 989–997. 22460505 10.1007/s10495-012-0717-2 PMC3413814

[21] Cheng HW , Liang YH , Kuo YL , Chuu CP , Lin CY , Lee MH , Wu ATH , Yeh CT , Chen EIT , Peng JW *et al* (2015) Identification of thioridazine, an antipsychotic drug, as an antiglioblastoma and anticancer stem cell agent using public gene expression data. Cell Death Dis 6, e1753–e1753. 25950483 10.1038/cddis.2015.77 PMC4669717

[22] Mu J , Huang W , Tan Z , Li M , Zhang L , Ding Q , Wu X , Lu J , Liu Y , Dong Q *et al* *.* (2017) Dopamine receptor D2 is correlated with gastric cancer prognosis. Oncol Lett 13, 1223–1227. 28454238 10.3892/ol.2017.5573 PMC5403577

[23] Khanna P , Komossa K , Rummel‐Kluge C , Hunger H , Schwarz S , El‐Sayeh HG and Leucht S (2013) Aripiprazole versus other atypical antipsychotics for schizophrenia. Cochrane Database Syst Rev 68, CD006569. 10.1002/14651858.CD006569.pub4 23450570

[24] Mailman RB and Murthy V (2010) Third generation antipsychotic drugs: partial agonism or receptor functional selectivity? Curr Pharm Des 16, 488–501. 19909227 10.2174/138161210790361461 PMC2958217

[25] Suzuki S , Okada M , Kuramoto K , Takeda H , Sakaki H , Watarai H , Sanomachi T , Seino S , Yoshioka T and Kitanaka C (2016) Aripiprazole, an antipsychotic and partial dopamine agonist, inhibits cancer stem cells and reverses chemoresistance. Anticancer Res 36, 5153–5161. 27798875 10.21873/anticanres.11085

[26] Kim MS , Yoo BC , Yang WS , Han SY , Jeong D , Song JM , Kim KH , Aravinthan A , Kim JH , Kim J‐H *et al* (2018) Src is the primary target of aripiprazole, an atypical antipsychotic drug, in its anti‐tumor action. Oncotarget 9, 5979–5992. 29464048 10.18632/oncotarget.23192 PMC5814188

[27] Koppel J , Jimenez H , Adrien L , Greenwald BS , Marambaud P , Cinamon E and Davies P (2016) Haloperidol inactivates AMPK and reduces tau phosphorylation in a tau mouse model of Alzheimer's disease. Alzheimers Dement 2, 121–130. 10.1016/j.trci.2016.05.003 PMC5644277 29067299

